# New Alzheimer Amyloid β Responsive Genes Identified in Human Neuroblastoma Cells by Hierarchical Clustering

**DOI:** 10.1371/journal.pone.0006779

**Published:** 2009-08-26

**Authors:** Markus Uhrig, Carina Ittrich, Verena Wiedmann, Yuri Knyazev, Annette Weninger, Matthias Riemenschneider, Tobias Hartmann

**Affiliations:** 1 Center for Molecular Biology of the University of Heidelberg (ZMBH), Heidelberg, Germany; 2 Institute for Neurobiology and Neurodegeneration, Saarland University, Homburg/Saar, Germany; 3 German Cancer Research Center (DKFZ), Heidelberg, Germany; 4 Klinik für Psychiatrie und Psychotherapie, Saarland University, Homburg/Saar, Germany; Tel Aviv University, Israel

## Abstract

Alzheimer's disease (AD) is characterized by neuronal degeneration and cell loss. Aβ_42_, in contrast to Aβ_40_, is thought to be the pathogenic form triggering the pathological cascade in AD. In order to unravel overall gene regulation we monitored the transcriptomic responses to increased or decreased Aβ_40_ and Aβ_42_ levels, generated and derived from its precursor C99 (C-terminal fragment of APP comprising 99 amino acids) in human neuroblastoma cells. We identified fourteen differentially expressed transcripts by hierarchical clustering and discussed their involvement in AD. These fourteen transcripts were grouped into two main clusters each showing distinct differential expression patterns depending on Aβ_40_ and Aβ_42_ levels. Among these transcripts we discovered an unexpected inverse and strong differential expression of neurogenin 2 (*NEUROG2*) and *KIAA0125* in all examined cell clones. C99-overexpression had a similar effect on *NEUROG2* and *KIAA0125* expression as a decreased Aβ_42_/Aβ_40_ ratio. Importantly however, an increased Aβ_42_/Aβ_40_ ratio, which is typical of AD, had an inverse expression pattern of *NEUROG2* and *KIAA0125*: An increased Aβ_42_/Aβ_40_ ratio up-regulated *NEUROG2*, but down-regulated *KIAA0125*, whereas the opposite regulation pattern was observed for a decreased Aβ_42_/Aβ_40_ ratio. We discuss the possibilities that the so far uncharacterized *KIAA0125* might be a counter player of *NEUROG2* and that *KIAA0125* could be involved in neurogenesis, due to the involvement of *NEUROG2* in developmental neural processes.

## Introduction

Amyloid beta precursor protein (*APP*), presenilin 1 (*PS1*), presenilin 2 (*PS2*) and apolipoprotein E (*APOE*) have been associated with AD [1] and further susceptibility genes are expected to exist. The amyloid cascade hypothesis suggests that Aβ_42_ accumulation is the primary event in the pathogenesis of AD. Aβ_42_, two amino acids (isoleucine and alanine) longer than Aβ_40_, is more prone to aggregate and expected to trigger the pathological cascade in AD [2], [3]. APP is cleaved by *β*-secretase resulting in the generation of the C-terminal fragment C99, which is further cleaved by the *γ*-secretase complex generating different Aβ species. By overexpressing C99 wildtype and C99 mutations, known to generate different Aβ_42_/Aβ_40_ levels [4], [5] in human neuroblastoma cells, we obtained information about the genome-wide gene regulation by using whole genome microarrays.

We unexpectedly identified *NEUROG2* and *KIAA0125* as inversely regulated by an altered Aβ_42_/Aβ_40_ ratio. Remarkably, an increased Aβ_42_/Aβ_40_ ratio that is typical of AD inverted the expression pattern of *NEUROG2* and *KIAA0125* that was observed for a decreased ratio. Importantly, for a decreased Aβ_42_/Aβ_40_ ratio *NEUROG2* and *KIAA0125* were the most differentially expressed transcripts out of approximately 40,000 tested.

Neurogenesis in AD is a controversial topic. In hippocampi of patients with AD [6], where it may produce cells to replace neurons lost in the disease [7], neurogenesis has been reported to be enhanced [8]. This could be reproduced in a transgenic mouse model [9] in which *APP* mutations led to increased incorporation of bromodeoxyuridine (BrdU) and enhanced expression of immature neuronal markers in two neuroproliferative regions: The subventricular zone and the dentate gyrus. In contrast to this, neurogenesis has been reported to be decreased in mouse models for AD [10], [11]. *NEUROG2* plays an essential role in the development of the dentate gyrus of the hippocampus [12], which is the central structure for learning and memory processes. The impairment of neurogenesis in a mouse model exhibiting progressive amyloid deposition was reflected by a reduction in the number of neural stem cells, progenitor cells and neuroblasts in the dentate gyrus [13]. Our work provides a possible mechanism of how neurogenesis might be influenced, namely by the previously undiscovered inverse regulation of *NEUROG2* and *KIAA0125* caused by Aβ.

## Results

We investigated the transcriptomic response to a changed Aβ_42_/Aβ_40_ ratio in human neuroblastoma cells. Constructs encoding the C-terminal part of APP (C99) were used to transfect human neuroblastoma cells in order to overexpress C99 [4], [5]. Subsequently, overexpressed C99 was processed by *γ*-secretase releasing several proportions of Aβ_42_ and Aβ_40_. To modify the Aβ_42_/Aβ_40_ ratio we made use of two well established constructs [4], [5]. The single point mutation I45F in C99 results in a strong overproduction of Aβ_42_ and a concomitant loss in Aβ_40_. The C99 point mutation V50F results in almost exclusive Aβ_40_ production. Total Aβ production from these constructs was similar in all clones and the clones used were expression level matched.

### SH-SY5Y cells overproduced different Aβ_42_ and Aβ_40_ levels

**Figure 1:**
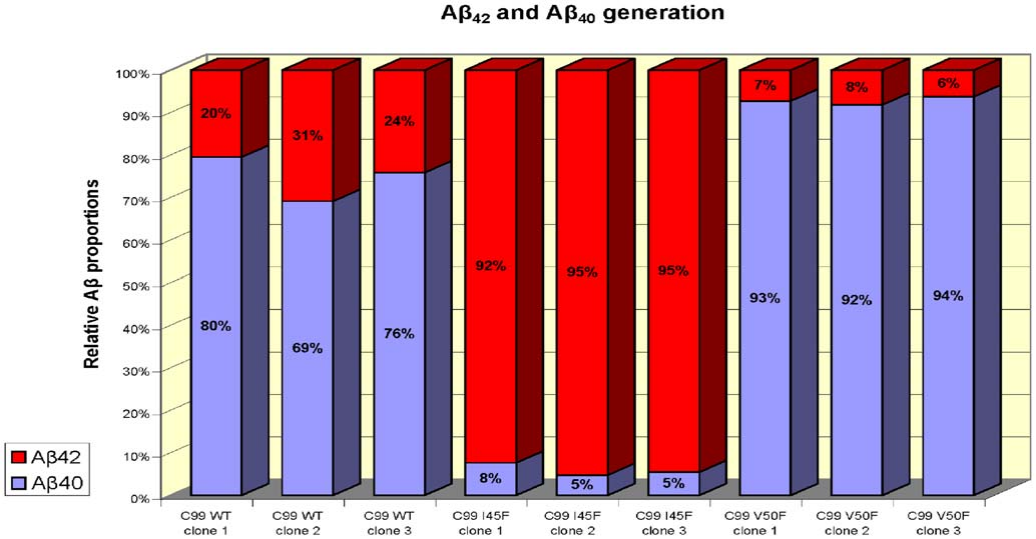
ELISA of Aβ_40_ and Aβ_42_ peptides from conditioned media of SH-SY5Y cells overexpressing C99. Aβ_40_ and Aβ_42_ were measured by ELISA. C99 was intracellularly cleaved, generating different amounts of Aβ_42_ and Aβ_40_ in C99WT, C99I45F and C99V50F. As was expected [4], [5], C99I45F transfected cells generated large amounts of Aβ_42_ and low levels of Aβ_40_ resulting in a large Aβ_42_/Aβ_40_ ratio, whereas the opposite regulation pattern was detected for C99V50F transfected cells. Mock-transfected cells only produced very low (endogenous) levels of Aβ_42_ and Aβ_40_, hence, their Aβ levels were not detectable, because they were close to the detection limit of the ELISA.

The human neuroblastoma cell line SH-SY5Y was stably transfected with constructs coding for the APP C-terminal fragment C99WT, moreover with constructs bearing the point mutations C99I45F and C99V50F and the vector control (mock-transfected, negative control). The point mutations were utilized to shift the Aβ_42_/Aβ_40_ ratio in either direction. As was described in detail [4], [5] C99V50F and C99I45F had an opposite effect on the Aβ peptides generated: As compared to the C99WT-transfected cells, the C99V50F-transfected cells expressed more Aβ_40_ and less Aβ_42_, whereas the C99I45F-transfected cells expressed more Aβ_42_ and less Aβ_40_ (Fig. 1).

**Figure 2:**
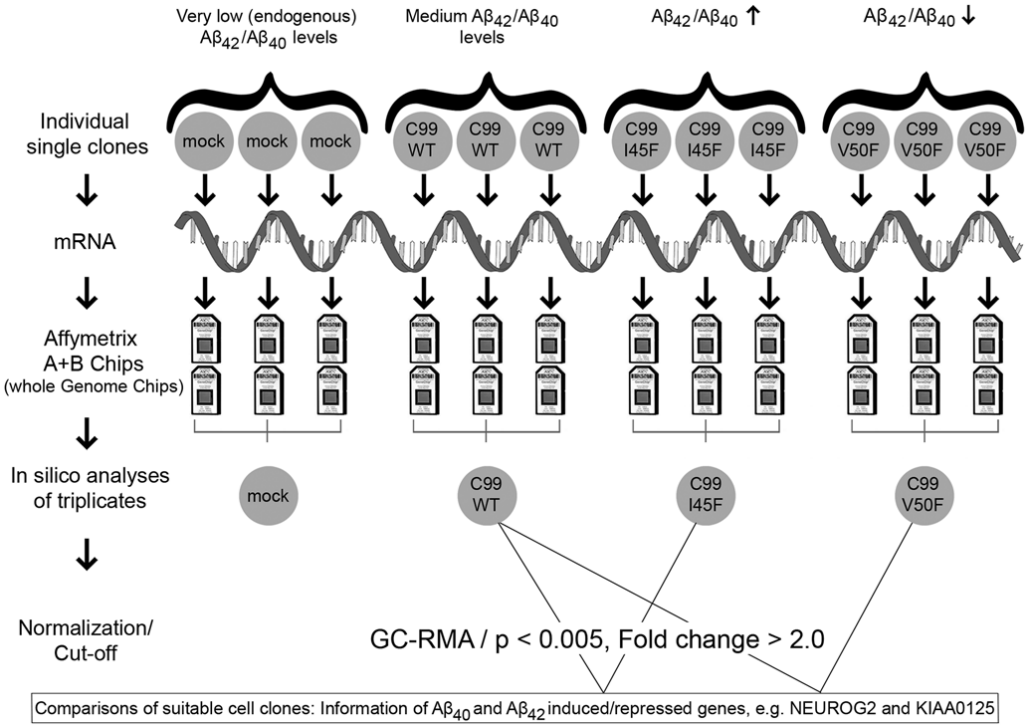
Experimental set up. Independent cell clones of the human neuroblastoma cell line SH-SY5Y, generating different amounts of Aβ_42_ and Aβ_40_, were used for whole genome transcription analysis. Total-RNA was extracted from the cells, converted into cDNA, followed by conversion into cRNA (in the scheme simplified presented as m-RNA). The cRNA was hybridized onto the Chips, washed, scanned and the scanned images were used for data analysis. The means of triplicates (n = 3 per group) were calculated and the groups were compared in order to obtain information about the effects of Aβ_42_/Aβ_40_. C99WT, producing medium Aβ_42_/Aβ_40_ levels, was compared with C99I45F (high Aβ_42_, low Aβ_40_ levels) and with C99V50F (low Aβ_42_, high Aβ_40_ levels). These comparisons resulted in *A*β*-specific* information. Comparisons between C99WT, C99I45F, C99V50F and mock-transfected cells resulted in information about effects caused by C99-overexpression *combined* with Aβ_42_/Aβ_40_ effects, since C99 as well as Aβ_42_/Aβ_40_ were overproduced compared to the mock-control in which only very low (endogenous) amounts of C99 and Aβ_42_/Aβ_40_ were present (Table 1).

Single independent cell clones (C99WT, C99V50F, C99I45F, mock-transfected negative control, n = 3 per group) were selected and used for whole genome microarray analysis. HG-U133 A and B microarrays (Affymetrix) were used (unprocessed and processed microarray data can be accessed via the ArrayExpress database, accession number E-MEXP-1913). Replicates from different *independent* clones were prepared and hybridized to the microarrays on different days. This procedure increased data variation between replicates, but more importantly, is expected to have increased accuracy (Fig. 2).

**Table 1:** Comparisons between cell clones and information obtained about C99 and Aβ-effects.

Comparisons	Effects
C99WT/mock	C99 overexpression
C99I45F/mock	C99 overexpression+Aβ_42_ overproduction
C99V50F/mock	C99 overexpression+Aβ_40_ overproduction
C99I45F/C99WT	Aβ_42_ overproduction
C99V50F/C99WT	Aβ_40_ overproduction

### Hierarchical clustering of Alzheimer Amyloid β Responsive Genes

**Figure 3:**
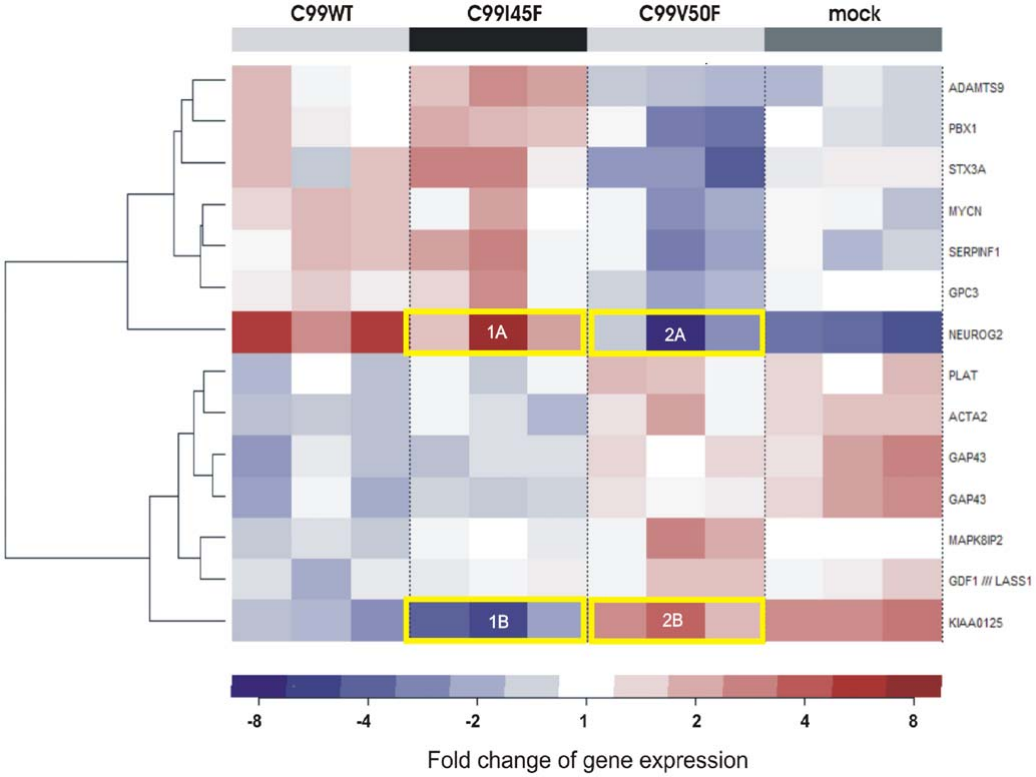
Hierarchical clustering of transcripts according to their expression values. Gene expression values of three *independent* replicates of C99WT, C99I45F, C99V50F and mock-transfected cells were clustered in order to obtain information about expression patterns. Two main clusters were identified. An inverse Aβ_42_/Aβ_40_ ratio resulted in an inverse *NEUROG2/KIAA0125* regulation. Genes were clustered with the Manhattan metric as distance between the centered expression profiles and complete linkage as distance between the clusters. GC-RMA normalized m-RNA levels were used. This procedure revealed a so far unknown correlation between *NEUROG2* and the previously uncharacterized *KIAA0125*: *NEUROG2* and *KIAA0125* were inversely regulated. Here, no baseline experiment was defined. Instead, for each probe set the mean over all chips was calculated and was subtracted from every single value (centering of data). The fold change refers to the mean of all expression values: *NEUROG2* (marked as 1A in Fig. 3) is upregulated approximately 8 fold (dark red colour) compared to the average expression of all chips appearing in Fig. 3. The average expression of all chips is regarded as a suitable baseline in order to see overall gene regulation in all cell clones. The yellow frames indicate the inverse regulation of *NEUROG2* and *KIAA0125* in the cells expressing inverse Aβ_42_/Aβ_40_ ratios: C99I45F (Aβ_42_/Aβ_40_↑) up-regulated *NEUROG2*, but down-regulated *KIAA0125*, whereas C99V50F (Aβ_42_/Aβ_40_↓) down-regulated *NEUROG2*, but up-regulated *KIAA0125* in all triplicates. Triplicates were derived from *independent* single clones so that clonal effects were highly unlikely. Interestingly, the replicate in which the strongest up-regulation was observed (1A) also showed the strongest down-regulation for *KIAA0125* (1B) and vice versa (2A and 2B).

In order to investigate the overall gene regulation and to discover novel expression patterns, all tested cell clones were used for cluster analysis. Hierarchical clustering is used to group genes according to common properties, for instance their expression levels. With this information one can gain insight into relationships between genes or their proteins. Certain expression patterns represent common regulatory processes, which can be a sign of putative functional relationships. To obtain insight into the gene regulation in all cell clones, gene expression values of three *independent* replicates of C99WT, C99I45F, C99V50F and mock-transfected cells were clustered (Fig. 3).

The cluster analysis revealed a previously unknown inverse regulation of *NEUROG2* and *KIAA0125* in consequence of an inverse Aβ_42_/Aβ_40_ ratio. Interestingly, both genes were the *most* differentially expressed ones and importantly, they were inversely regulated in *all* measured cell clones.

To focus specifically on Aβ effects, it was essential to compare suitable cell clones. While comparisons of C99WT, C99I45F and C99V50F with mock-transfected cells provided information about C99-overexpression effects, comparisons of C99I45F and C99V50F with C99WT provided information about Aβ effects (Table 1). Our goal was to obtain information about Aβ_42_/Aβ_40_ effects, therefore we first concentrated on C99V50F, generating a decreased Aβ_42_/Aβ_40_ ratio, and compared it to C99WT generating medium Aβ_42_/Aβ_40_ levels. Whole genome expression profiling of these cell clones resulted in a gene list in which the genes were sorted according to their differential expression levels (Table 2). The first most up-regulated and first most down-regulated genes were then further investigated: We examined how these two genes were expressed when the Aβ_42_/Aβ_40_ ratio was increased. Since an increased Aβ_42_/Aβ_40_ ratio, as this is the case with C99I45F-transfected cells, is characteristic of AD, this approach was expected to provide essential information about the pathomechanism of AD.

**Table 2:** Most up and down-regulated transcripts derived from the comparison C99V50F/C99WT1 (Aβ_42_/Aβ_40_↓).

Position	Probe set ID	p-value	Fold change C99V50F/C99WT1 Aβ_42_/Aβ_40_↓	Gene symbol	Gene title	Chromo-somal location
**1**	**206478_at**	**0.00142**	**5.3**	***KIAA0125***	**KIAA0125**	**chr14q32.33**
2	208603_s_at	0.00191	2.5	*MAPK8IP2*	mitogen-activated protein kinase 8 interacting protein 2	chr22q13.33
3	200974_at	0.00115	2.2	*ACTA2*	actin, alpha 2, smooth muscle, aorta	chr10q23.3
4	216963_s_at	0.00449	2.2	*GAP43*	growth associated protein 43	chr3q13.1-q13.2
5	201860_s_at	0.00477	2.1	*PLAT*	plasminogen activator, tissue	chr8p12
6	206397_x_at	0.00269	2.1	*GDF1*	growth differentiation factor 1	chr19p12
7	204471_at	0.00190	2.1	*GAP43*	growth associated protein 43	chr3q13.1-q13.2
7	220287_at	0.00110	−2.0	*ADAMTS9*	a disintegrin-like and metalloprotease (reprolysin type) with thrombospondin type 1 motif, 9	chr3p14.3-p14.2
6	209220_at	0.00089	−2.5	*GPC3*	glypican 3	chrxq26.1
5	212148_at	0.00288	−2.8	*PBX1*	pre-B-cell leukemia transcription factor 1	chr1q23
4	209757_s_at	0.00416	−3.0	*MYCN*	v-myc myelocytomatosis viral relatedoncogene, neuroblastoma derived (avian)	chr2p24.1
3	202283_at	0.00362	−3.1	*SERPINF1*	serine proteinase inhibitor,clade F (alpha-2 antiplasmin, pigmentepithelium derived factor), member 1	chr17p13.1
2	209238_at	0.00268	−3.7	*STX3A*	syntaxin 3A	chr11q12.1
**1**	**215632_at**	**0.00166**	**−12.5**	***NEUROG2***	**neurogenin 2**	**chr4q25**

Table 2 Most up and down-regulated transcripts derived from the comparison C99V50F/C99WT1 (Aβ_42_/Aβ_40_↓; n = 3 per group; p < 0.005, *un*adjusted p-values; C99WT1 was the baseline experiment). The unspliced *GAP43* and its spliced isoform were detected by two different probe sets (204471_at and 216963_s_at).

### Neurogenin 2 was strongly down-regulated, whereas *KIAA0125* was strongly up-regulated in consequence of a decreased Aβ_42_/Aβ_40_ ratio

**Figure 4:**
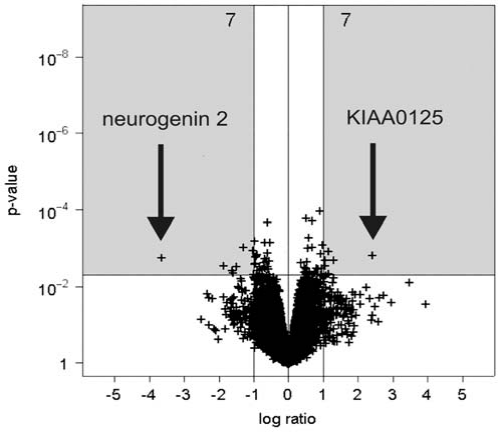
Volcano plot of up and down-regulated transcripts derived from the comparison C99V50F/C99WT1 (Aβ_42_/Aβ_40_↓, n = 3 per group, p < 0.005, C99WT1 was the baseline experiment). A fold change of gene expression≥2.0 (|log_2_ ratio|≥1) was regarded as biologically meaningful. 14 significantly differentially expressed transcripts were identified (seven up-regulated and seven down-regulated ones, see grey boxes).

The cells expressing a decreased Aβ_42_/Aβ_40_ ratio (C99V50F) were compared to the cells transfected with the wildtype construct (C99WT). C99V50F expresses a smaller Aβ_42_/Aβ_40_ ratio compared to the Aβ_42_/Aβ_40_ ratio in C99WT (Fig. 1). Fourteen significantly dysregulated transcripts (seven up-regulated and seven down-regulated ones) were identified with *NEUROG2* and *KIAA0125* being the most prominent (Fig. 4). The corresponding probe set identification numbers (Probe set IDs), p-values, fold changes, gene symbols, gene titles and chromosomal locations are listed in Table 2.

### Real-time PCR validated the inverse expression of *NEUROG2* and *KIAA0125* and revealed a correlation to relative Aβ_42_ levels

**Figure 5:**
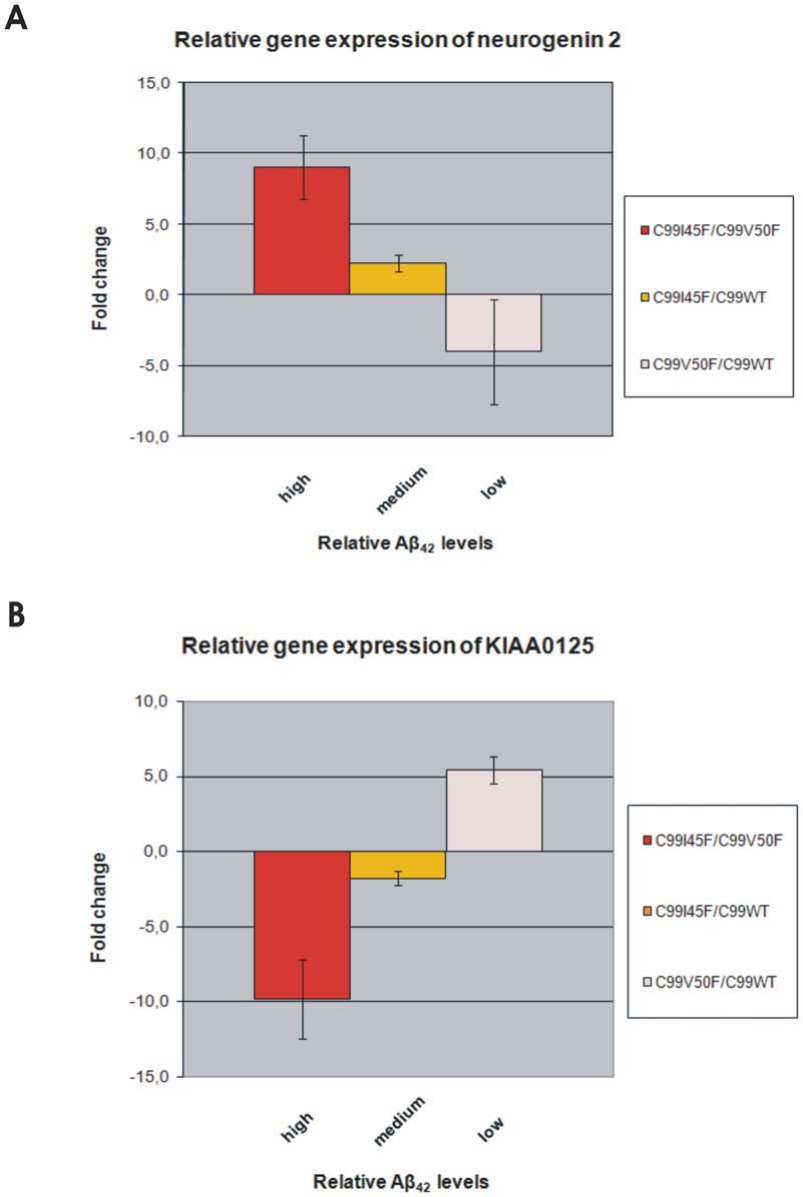
Relative gene expression of *NEUROG2* and *KIAA0125* measured by real-time PCR and compared to relative Aβ_42_ levels. Fig. 5A and 5B show an almost linear relationship of *NEUROG2/KIAA0125* expression and relative Aβ_42_ levels. It is noteworthy that these relationships are in opposite directions: While *NEUROG2* expression increases with increasing relative Aβ_42_ levels, *KIAA0125* expression decreases with increasing relative Aβ_42_ levels. Importantly, the same regulation pattern was confirmed by real-time PCR as previously observed by microarray analysis: The stronger the *NEUROG2 up*-regulation in certain cell clones (Fig. 5A), the stronger was the *KIAA0125 down*-regulation in the same cell clones (Fig. 5B) and vice versa. Total-RNA was originated from the same clones as the ones used for the microarrays. This total-RNA was converted into cDNA and used for real-time PCR. Cyclophilin A expression was used for normalisation. Error bars represent the standard error of the mean (S.E.M.) of three independent cell clones.

*NEUROG2* and *KIAA0125* transcription levels were measured by quantitative real-time PCR and compared to relative Aβ_42_ levels (Fig. 5A, 5B).

### *NEUROG2* gene expression correlates positively with relative Aβ_42_ levels as demonstrated by real-time PCR

Relative Aβ_42_ levels were ranked from high to low and plotted against the differential *NEUROG2* expression (Fig. 5A). Here, we demonstrated that *NEUROG2* expression increased together with increasing relative Aβ_42_ levels. *NEUROG2* showed the strongest up-regulation (9.0 fold, S.E.M. = 2.3) for mutant C99I45F versus C99V50F, for which high relative Aβ_42_ levels were generated. *NEUROG2* showed up-regulation (2.3 fold, S.E.M. = 0.6) for the comparison C99I45F versus C99WT; for this comparison medium relative Aβ_42_ levels were generated. *NEUROG2* was down-regulated 4.0 fold (S.E.M. = 3.7) in mutant C99V50F compared to C99WT; for this comparison low relative Aβ_42_ levels were generated.

### *KIAA0125* gene expression correlates negatively with relative Aβ_42_ levels as demonstrated by real-time PCR

Relative Aβ_42_ levels were ranked from high to low and plotted against the differential *KIAA0125* expression (Fig. 5B). *KIAA0125* showed an inverse expression pattern to that of *NEUROG2* in response to increased relative Aβ levels. *KIAA0125* showed the strongest down-regulation (9.8 fold, S.E.M. = 2.6) for the comparison of mutant C99I45F versus C99V50F, for which the highest relative Aβ_42_ levels were generated. *KIAA0125* was down-regulated 1.8 fold (S.E.M. = 0.5) for the comparison C99I45F versus C99WT (medium relative Aβ_42_ levels) and it was up-regulated 5.4 fold (S.E.M. = 0.9) in mutant C99V50F compared to C99WT, for which the lowest relative Aβ_42_ levels were generated.

### *NEUROG2* and *KIAA0125* are inversely regulated in all tested cell clones

Comparing the gene expression of *NEUROG2 and KIAA0125*, measured by real-time PCR, confirmed the results revealed by microarray analysis: The stronger the *NEUROG2 up*-regulation in certain cell clones (Fig. 5A), the stronger is the *KIAA0125 down*-regulation in the same cell clones (Fig. 5B) and vice versa. C99-overexpression had a similar effect on *NEUROG2* and *KIAA0125* expression as a decreased Aβ_42_/Aβ_40_ ratio. Importantly however, an increased Aβ_42_/Aβ_40_ ratio, which is typical of AD, had an inverse expression pattern of *NEUROG2* and *KIAA0125*: A decreased Aβ_42_/Aβ_40_ ratio down-regulated *NEUROG2*, but up-regulated *KIAA0125*, whereas an increased Aβ_42_/Aβ_40_ ratio up-regulated *NEUROG2*, but down-regulated *KIAA0125*.

## Discussion

*Neurog 2* (synonym: *Math4A*) and the so far uncharacterized *KIAA0125* were the most extremely and inversely regulated genes in consequence of a decreased Aβ_42_/Aβ_40_ ratio: While *KIAA0125* was the most up-regulated gene, *NEUROG2* was the most down-regulated one. However, for an increased Aβ_42_/Aβ_40_ ratio, which is typical of AD, the expression pattern was inverted: *NEUROG2* was the second most up-regulated gene, whereas *KIAA0125* was strongly down-regulated. Bearing in mind that 40.000 transcripts were analyzed here, finding such a regulation pattern merely by chance is rather unlikely and argues for a *specific* effect mediated by the Aβ_42_/Aβ_40_ ratio.

Analyzing large data sets can increase the error in significance testing (problem of multiplicity). To keep that error small we decided for a cut-off of p < 0.005. We calculated adjusted p values to control the false discovery rate (FDR) by the method of Benjamini & Hochberg (shown in the Supplementary Information, Table S1). For several of the comparisons investigated, we got FDR-adj usted p values less than e.g. 0.05 (C99I45F vs. mock, C99V50F vs. mock, C99WT vs. mock). This doesn't hold true for C99V50F vs. C99WT. Hence, we chose a cut off for the *un*adjusted p values being aware of the limitations of the resulting gene list. To exclude the possibility of false positives, data can be validated with another method. This was done by real-time PCR for the genes *KIAA0125* and *NEUROG2*, which verified the results of the microarray analysis.

For analyzing the effects of Aβ_42_ and Aβ_40_, the direct comparison between C99I45F or C99V50F and C99WT is more suitable than the comparison between C99I45F or C99V50F and the mock-control. However, the comparison between the Aβ-overproducing mutants C99I45F or C99V50F and the mock-control also deliver interesting information, namely information about Aβ effects *combined* with C99 effects (Table 1). Hence, we put these data into the Supplementary Information (Table S1).

Intriguingly, *KIAA0125* (on chromosome 14q32.33) is localized close to the presenilin 1 (*PS1*) locus (chr14q24.3). The *KIAA0125* gene localizes to the immunoglobulin heavy chain locus (IGH@) on chromosome 14. *KIAA0125* has been suggested to be a gene with putative protein-coding properties (hypothetical protein: LOC9834). The function of this putative protein has not been determined yet. Interestingly, a sequence of 76–78 nucleotides was found repeated 6 times in the untranslated region of *KIAA0125* [14] possibly arguing for a regulatory function. The calculated molecular weight is expected to be 8.1 kDa (according to the human protein reference database) or 7.7 kDa (according to the UniProt/Swiss-Prot databases). Provided the mRNA is actually translated, the complete coding sequence is expected to be a peptide with 76 amino acids (according to the Uniprot database, based on the nucleotide sequence).

*NEUROG2* is a member of the neurogenin subfamily of basic helix-loop-helix (bHLH) transcription factors that play an important role in neurogenesis. During mouse neurogenesis, *NEUROG2* and *NEUROG1* are expressed in distinct progenitor populations in the central and peripheral nervous systems [15]. Yan et al. observed that in the developing chick retina, *NEUROG2* was expressed in a subpopulation of proliferating progenitor cells [16]. Scardigli et al. hypothesized that *NEUROG2* is both responsive to, and a regulator, of genetic pathways that specify neuronal fates in the ventral spinal cord [17]. It has been shown that the development of mesencephalic dopamine neurons is severely compromised in *NEUROG2-*null mutant mice. *NEUROG2* is required for the differentiation of ventricular zone progenitors into postmitotic dopaminergic neuron precursors in the intermediate zone. It was concluded that *NEUROG2* is required for the development of midbrain dopaminergic neurons [18]. *NEUROG2* was immuno-histochemically detected in a certain cycling population during G1 phase and was further restricted during G2-M phases to the subventricular zone-directed population. *NEUROG2* may further be involved in the asymmetric cell divisions of progenitor cells [19]. Moreover, it has been reported that inhibition of proneural bHLH factors, like NEUROG2, in cortical progenitors promotes the formation of astrocytes [20].

A changed Aβ_42_/Aβ_40_ ratio is typically found in familial Alzheimer's disease (FAD) and we used the same terminology here. It should be noted however, that the basic increases or decreases of the individual Aβ species levels might have biological effects, which can be independent of the Aβ ratio [21], [22]. Indeed, based on absolute Aβ species levels (Supplementary Information, Table S2), the unbiased cluster analysis (Fig. 3) would be in agreement with an Aβ_42_ effect. This is reflected by the grouping of mock and C99V50F in one main cluster and C99WT and C99I45F in another one, which is in agreement with the extent of absolute Aβ_42_ levels.

We propose the following property/function to *KIAA0125*: Firstly, it can be speculated that *KIAA0125* may act as an antagonist of *NEUROG2*, secondly, that inverting the Aβ_42_/Aβ_40_ ratio also inverts the expression of *NEUROG2*/*KIAA0125*, thirdly, it can further be speculated that *KIAA0125* might play a role in neurogenesis, maybe in preventing the generation of dopaminergic neurons or it could also be involved in inducing astrocytosis.

It had not escaped our notice that also other genes are clustered in similar ways like *KIAA0125* (for instance *GAP43*, see Fig. 3). However, these genes do not reach such extreme differential expression values like *KIAA0125*. Though, for such genes an important relationship to *NEUROG2* may also exist. Moreover, it has to be taken into consideration that further genes exist, which do not pass our cut-off criteria for significance (p < 0.005); for some of these genes a relationship similar to that of *KIAA0125* cannot be excluded.

Our work has not only been restricted to *NEUROG2* and *KIAA0125*, but provides further information about the regulation of several transcripts involved in Aβ induced gene expression. While differential expression for *NEUROG2 and KIAA0125* was validated by real-time PCR, any remaining genes listed in Table 2 were not validated by another method. However, using a rather stringent cut-off for significance (p < 0.005; *un*adjusted p-values) provided more confidence in these data than using the frequently used threshold of p < 0.05.

Interestingly, the *KIAA0125* regulation was found to be similar to the one of growth associated protein 43 (*GAP43*) and plasminogen activator, tissue (*PLAT*). GAP43 has been termed a ‘growth’ or ‘plasticity’ protein because it is expressed at high levels in neuronal growth cones during development and axonal regeneration. It is considered to be a crucial component of an effective regenerative response in the nervous system. Phosphorylation of this protein by protein kinase C is specifically correlated with certain forms of synaptic plasticity. The fact that *GAP43* was up-regulated in consequence of a decreased Aβ_42_/Aβ_40_ ratio, but not in response to an increased one, is in line with the aberrant *GAP43* gene expression that has been observed in AD [23]. Furthermore, it has been demonstrated that treatment of neuronal cultures with Aβ_40_ for four days dose-dependently increased *GAP43* levels and it has been suggested that Aβ_40_ may promote neurite formation in primary neuronal cultures [24].

Plasminogen activator, tissue (*PLAT*) is regarded as one of the top candidate genes in AD according to the Alzforum database (www.alzforum.org). It is one of the most prominent activators of fibrinolysis. Its up-regulation in consequence of a decreased Aβ_42_/Aβ_40_ ratio (whereas an increased ratio did not up-regulate *PLAT*) may contribute to enhanced fibrinolysis. This may offer an explanation for the increased tendency of having strokes in AD patients because for an increased Aβ_42_/Aβ_40_ ratio, typical of AD, this putative protective up-regulation of *PLAT* might be missing. Most interestingly, *PLAT* was found to be down-regulated 3.3 fold (p = 0.0037) in the brains of Down's syndrome patients (see supplemental information published in [25]), who also have a greater prevalence of strokes [26] and in which Aβ_42_ levels are increased due to a gene dosage effect caused by triplication of the *APP* gene localized on chromosome 21 (trisomy 21). These observations argue for a negative correlation between *PLAT* expression and the Aβ_42_/Aβ_40_ ratio *in vitro* and *in vivo*.

Fibrin, the end product of blood coagulation, can be converted into soluble fragments (fibrinolysis). Plasmin, a protease, converts fibrin into soluble fragments by cleavage. The serine proteinase inhibitor, clade F, member 1 (*SerpinF1*, α-2 anti-plasmin) is an anti-plasmin and it was down-regulated 3.1 fold (p = 0.00362) for a decreased Aβ_42_/Aβ_40_ ratio, whereas it was not differentially expressed when the Aβ_42_/Aβ_40_ was increased. It can be speculated that more plasmin may be available, which in turn could accelerate fibrinolysis (for a decreased Aβ_42_/Aβ_40_ ratio).

Actin, alpha 2, smooth muscle, aorta (ACTA2) is one of six different actin isoforms. Actins are highly conserved proteins that are involved in cell motility and cell structure. They are ubiquitous proteins involved in the formation of filaments that are major component of the cytoskeleton. Interaction with myosin provides the basis of muscular contraction and many aspects of cell motility. *ACTA2* was the third most strongly up-regulated gene for Aβ_42_/Aβ_40_↓, but it was not differentially expressed for Aβ_42_/Aβ_40_↑. It was co-regulated with *GAP43*, *PLAT, GDF1* and *MAPK8IP2*. ACTA2 has been described as being involved in vascularisation and vascular branching [27], [28]. Furthermore, impaired vascular contractility and blood pressure homeostasis in smooth muscle α-actin null mice have been observed [29]. The major function of vascular smooth muscle cells is contraction to regulate blood pressure and flow [30]. It can be speculated that upregulation of *ACTA2* in consequence of a decreased Aβ_42_/Aβ_40_ ratio is a mechanism that might also occur in smooth muscle cells, which in turn could lead to improved vascularisation and could regulate blood pressure. This positive effect on vascularisation and blood pressure might be missing for the AD-typical increased Aβ_42_/Aβ_40_ ratio where no differential *ACTA2* expression was observed. This could help to understand why high systolic blood pressure is a risk factor for AD.

Due to its involvement in neurotransmitter release, Syntaxin 3A dysregulation was of special interest. Syntaxin 3A was found to be inversely regulated: It was weakly up-regulated in consequence of an increased Aβ_42_/Aβ_40_ ratio (p = 0.159, fold change 1.4), whereas it was down-regulated in consequence of a decreased one (p = 0.00268, fold change = −3.7). Syntaxins interact with synaptotagmins and are responsible for membrane fusions of transmitter containing vesicles. Synaptotagmin XIII was up-regulated in consequence of an increased Aβ_42_/Aβ_40_ ratio (data not shown) and could possibly be an interaction partner for syntaxin 3A. It can be speculated that neurotransmitter release is influenced by dysregulation of syntaxin 3A.

ADAMTS9 (a disintegrin-like and metalloprotease, reprolysin type, with thrombospondin type 1 motif, 9) belongs to the ADAMTS family. Members of the ADAMTS family have been implicated in the cleavage of proteoglycans, the control of organ shape during development and the inhibition of angiogenesis. ADAMTS9 is a secreted, cell-surface-binding metalloprotease that cleaves the proteoglycans versican and aggrecan and binds Zn^2+^ ions [31]. Unlike most precursor proteins, the ADAMTS9 zymogen (pro-ADAMTS9) is resistant to intracellular processing. Instead, pro-ADAMTS9 is processed by furin at the cell surface. It is suggested that, unlike other metalloproteases, furin processing of the ADAMTS9 propeptide reduces its catalytic activity [32]. Observations suggest that the propeptide is a key functional domain of ADAMTS9, mediating an unusual regulatory mechanism that may have evolved to ensure maximal activity of this protease at the cell surface. ADAMTS proteins are structurally homologous to ADAM proteins, but they also contain at least 1 C-terminal thrombospondin type 1 (TSP1) repeat and are secreted rather than membrane bound. ADAMTS9 was found to be up-regulated in consequence of an increased Aβ_42_/Aβ_40_ ratio and down-regulated as a result of a decreased one. This inverse regulation argues for a specific effect mediated by the Aβ_42_/Aβ_40_ ratio itself, because an inverse Aβ_42_/Aβ_40_ ratio led to an inverse regulation of this gene and may not be mediated by unspecific effects occasionally observed in microarray studies.

Taken together, we demonstrated that the expression levels of *KIAA0125* and *NEUROG2* were inversely regulated by an altered Aβ_42_/Aβ_40_ ratio: An increased Aβ_42_/Aβ_40_ ratio, which is typical of AD, up-regulated *NEUROG2* but down-regulated *KIAA0125, whereas* the opposite regulation pattern was observed for a decreased ratio. This might indicate a biological function for the so far uncharacterized *KIAA0125*: It may be speculated that *KIAA0125* could be involved in neurogenesis possibly by an action antagonistic to that of *NEUROG2*, due to the observed strict inverse regulation of both genes and the already established involvement of *NEUROG2* in developmental neural processes. Finally, our dataset provides information about the regulation of further Aβ dependent genes, which could turn out to be important in AD.

## Materials and Methods

### Cell line, cell culture and transfections

The human neuroblastoma cell line SH-SY5Y [33], [34] was cultured in 50% Minimum Essential Medium (MEM, Sigma) and 50% Nutrient Mixture F-12, HAM (Sigma), supplemented with 10% FBS (PAN), 1% L-Glutamin (Sigma) and 1% nonessential amino acid solution (Sigma) in a humidified atmosphere with 5% CO_2_. 70% confluent SH-SY5Y cells were transfected with the constructs mentioned below.

### Plasmids

Sequences coding for C99WT, C99I45F and C99V50F were cloned into a pCEP4 vector (Invitrogen). These plasmid constructs have been previously described [4], [5]. The empty vector pCEP4 (mock) was used as a negative control.

### Enzyme-linked immunosorbent assay (ELISA) of Aβ_42_ and Aβ_40_

Subconfluent cells were grown in 5 ml culture medium and conditioned for 48 h. Conditioned medium was collected, then Aβ_42_ and Aβ_40_ concentrations were measured by an enzyme-linked immunosorbent assay, following the manufacturer's recommendations. Measurements were carried out using a 96-Well MULTI-SPOT Human (6E10) Abeta Triplex Assay (MSD, Haass).

### Transcriptomics and data analysis

Microarray analysis was performed according to the Expression Analysis Technical Manual (Affymetrix) with minor modifications: Briefly, total RNA was extracted using the Qiashredder-Kit, RNase-free DNase set (Qiagen) and RNeasy Midi-columns (Qiagen). 20 µg of total RNA was reverse transcribed into cDNA by using the Superscript™ Double-Stranded cDNA Synthesis Kit (Invitrogen) and oligo(dT) primers (Proligo). 3.3 µl of purified cDNA was converted into cRNA using the BioArray™ High Yield™ RNA Labeling Kit (Enzo Life Sciences). Subsequently, 15 µg of purified cRNA was fragmented using the GeneChip® Eukaryotic Hybridization Control Kit (Affymetrix). 15 µg of fragmented cRNA was hybridized to whole genome HG-U133 A and HG-U133 B Chips. Chips were washed, stained, scanned and the quality of the created dat-file images was evaluated by using Gene Operating Software GCOS 1.2 and MAS 5.0 Software (Affymetrix). The sample quality was checked by using a Bioanalyzer 2100 (Agilent). The statistical analysis was carried out using the software package R, version 1.9.1 (R Development Core Team (2004) *R: A language and environment for statistical computing*. R Foundation for Statistical Computing, Vienna, Austria), together with libraries *gcrma* and *limma* of the Bioconductor Project, version 1.4 [35]. The data preprocessing steps, background-adjustment, normalization and computation of GC-RMA gene expression measures were performed according to Wu et al. (Wu Z; Irizarry RA; Gentleman R; Martinez-Murillo F; Spencer F (2004): A Model-Based Background Adjustment for Oligonucleotide Expression Arrays, Johns Hopkins University, Dept. of Biostatistics Working Papers, Working Paper 1). For the statistical analysis, empirical Bayes inference for linear models with the transfected neuroblastoma cell line SH-SY5Y (C99WT1, C99I45F, C99V50F, mock - with 3 replicates per group) was used [36]. Moderated *t*-statistics and corresponding p-values were calculated for the comparisons C99V50F vs. C99WT1. We used a threshold of 0.005 for the p-values and selected only those probe sets which showed a |log_2_ ratio|≥1. A hierarchical clustering (Manhattan metric+complete linkage) for the centered expression profiles over all experimental groups was performed for the presentation of probe sets selected.

### Quantitative real-time PCR and selection of an endogenous control for normalisation

Total-RNA was reverse transcribed into cDNA using random hexamer primers included in the High-Capacity cDNA Archive Kit (Applied Biosystems). This cDNA was amplified and measured by using TaqMan® Gene expression assays (Applied Biosystems). Cycling conditions were: 50°C for 2 min, 95°C for 10 min, followed by 40 cycles of 95°C for 15 s and 60°C for 1 min. Relative quantification was performed with the 2^−ΔΔ^C_T_ method. For normalisation, an endogenous control was selected out of 10 candidate controls using the TaqMan® Human Endogenous Control Plate (Applied Biosystems).

### Quality control of cells, target-RNA and arrays

Cells were checked for mycoplasma contamination. The 260 nm/280 nm ratio for total-RNA was between 1.9 and 2.1 for microarray experiments. Total-RNA and unfragmented cRNA was checked with a Bioanalyzer 2100 (Agilent). For total-RNA, two distinct bands (28 s and 18 s ribosomal RNA) were detected; the 28 s band was approximately twice as strong as the 18 s band. For unfragmented cRNA an accumulation of bands in the center of each lane, representing the different m-RNAs, was detected. For fragmented cRNA, bands, corresponding to a size of 35–200 bases, were detected. After scanning, array images were assessed by eye to confirm the absence of bubbles or scratches. The means of all chips are shown; the highest and lowest value is indicated in brackets. Target intensities of 100 (HG-U133 A Chip) and 20 (HG-U133 B Chip) were used. Only chips with equal target intensities were compared among each other. Scaling factors for A-chips were within acceptable limits 0.91 (0.8–1.4), as were background 75.1 (60.7–97.7), noise (rawQ) 2.7 (2.4–3.3) and number of present transcripts 51% (47.4–52.9%). 3′/5′ ratios for *GAPDH* and β-actin were confirmed to be within acceptable limits (*GAPDH*: 0.92 (0.79–1.81), β-actin: 1.26 (1.03–2.29), and BioB spike controls were found to be present on 100% of all the chips, with BioC, BioD and CreX also present in increasing intensity. Scaling factors for all B-chips were within acceptable limits 1.24 (0.9–1.6), as were background 63.87 (43.9–112), noise (raw Q) 2.6 (2.0–3.6) and number of present transcripts 30% (14.3–38,4%). 3′/5′ ratios for *GAPDH* and β-actin were confirmed to be within acceptable limits (*GAPDH*: 1.1 (0.88–2.03), β-actin: 1.3 (0.92–2.95), and BioB spike controls were found to be present on 95% of all the chips, with BioC, BioD and CreX also present in increasing intensity.

### Accession Number

Unprocessed and processed microarray data were deposited in the ArrayExpress database (http://www.ebi.ac.uk/microarray-as/ae/) under the accession number E-MEXP-1913.

## Supporting Information

Table S1 Supplementary Information, C99I45F vs mock, C99V50F vs mock, C99WT vs mock
(0.15 MB XLS)

Table S2 Supplementary Information, ELISA
(0.02 MB XLS)
